# Experiences of body image changes in Chinese hematopoietic stem cell transplantation survivors: A qualitative study

**DOI:** 10.1016/j.apjon.2025.100717

**Published:** 2025-05-08

**Authors:** Jia Yin Ruan, Lingxiang Lu, Wing Fai Yeung, Ying Qian, Mary-Ellen Brierley, Yim Wah MaK, Yiyu Zhuang

**Affiliations:** a New York University Rory Meyers College of Nursing, New York, United States; b Sir Run Run Shaw Hospital, Zhejiang University School of Medicine, Zhejiang, China; c School of Nursing, Hong Kong Polytechnic University, Hong Kong, China; d Melbourne School of Psychological Sciences, University of Melbourne, Victoria, Australia

**Keywords:** Cancer survivors, Graft-versus-host disease, Hematopoietic stem cell transplantation, Identity negotiation, Qualitative research, Survivorship

## Abstract

**Objective:**

This study aimed to explore the experiences of body image changes in hematopoietic stem cell transplantation survivors in China and to depict how those changes affected their perception of self and body.

**Methods:**

A qualitative descriptive approach was applied to provide a straight and comprehensive understanding of experiences in body image changes. Twenty hematopoietic stem cell transplantation survivors who underwent transplantation at a Chinese tertiary hospital were selected through purposeful sampling. Data were collected via semi-structured interviews and analyzed using conventional content analysis. Reporting adhered to the Consolidated Criteria for Reporting Qualitative Research Checklist.

**Results:**

Three themes and 11 subthemes were identified: (1) I am an inferior person (a distinctly weird appearance, an impaired body function, and depending on external resources for survival); (2) I am a person struggling to reconcile with my changed body (struggling to conceal or disclose my changed body, negotiating the impact of my changed body on my work identity, struggling to understand and care for my changed body, and catching up in rumination over my changed body); and (3) I am a brand-new person (having escaped from the gate of hell, enhanced appearance, optimized personality, possessed sublimated emotions).

**Conclusions:**

This qualitative study offers novel insights into the experiences of body image changes in patients with hematological cancers post-hematopoietic stem cell transplantation within the Chinese context. It also presents a journey toward accepting body image changes. Further research is warranted to explore the underlying mechanisms linking body image changes to shifts in self-identity, and the process of body image acceptance.

## Introduction

Hematological malignancies refer to cancers that affect the blood, bone marrow, and lymph nodes (e.g., acute leukemias and lymphoma). The curative treatment for many hematological conditions is hematopoietic stem cell transplantation (HSCT), which involves administering healthy hematopoietic stem cells to patients with dysfunctional or depleted bone marrow.^1^ Due to the high prevalence of hematological malignancies,^2^ the number of patients receiving HSCT is rapidly increasing worldwide.^3^ In 2018, a total of 93,105 HSCTs were performed across 89 countries, nearly doubling the figure from 2007, which reported 48,709 HSCTs.^4^ By 2019, 1.5 million HSCTs had been performed globally since 1957.^3^ A similar upward trend has been observed in China. Between 2020 and 2021, a total of 31,525 HSCTs were performed in China,^5^ and in the following two years (2022 and 2023), 39,918 HSCTs were carried out.^6^ With a reduction in cancer-related mortality and an improvement in outcomes for HSCT,^7^ the number of HSCT survivors has increased.

HSCT primarily consists of two types: allogeneic HSCT (allo-HSCT) and autologous HSCT (auto-HSCT). The former uses stem cells from an unrelated donor and offers the advantage of a lower risk of disease relapse, but it comes with an increased risk of treatment-related complications, including graft-versus-host disease (GVHD).^8^ The latter involves reinfusing stem cells from the patient's own hematopoietic stem cells; it reduces transplant-associated morbidity but increases the risk of disease recurrence.^8^ Regardless of the type of HSCT, patients typically experience changes in body image to varying extents during survivorship.^9^ This period begins after successful transplantation^10^^,^^11^ and is characterized by the patient being alive and free of disease recurrence.^10^ Survivorship can be further categorized into short-term (less than 1 year post-HSCT) and long-term (1 year or more post-HSCT, typically 2 years or more post-HSCT).^10^^,^^12^

Body image is a complex, multidimensional, and subjective construct that refers to a person's beliefs, attitudes, and perceptions about his or her physical appearance and bodily function.^13^ Drawing on Merleau-Ponty's phenomenology of embodiment,^14^ the body is not a passive object but the lived body—the subjective medium through which individuals perceive, relate to, and act within the world. From this perspective, body image is not merely a cognitive or visual representation, but a pre-reflective, embodied experience that forms the foundation of one's sense of self and presence. Medical treatments, such as HSCT, can disrupt this embodied continuity, fragmenting the habitual body schema and generating a sense of estrangement from the body. These disruptions may result in depression, identity loss, diminished self-esteem, reduced quality of life, and difficulties in social reintegration—especially when individuals struggle to reconcile their transformed bodies with their previous sense of normalcy.^15^, ^16^, ^17^

Under these circumstances, how do HSCT survivors experience changes in their body image? What influence do these changes have on their perception of self and body? These questions can be explored by investigating the experiences of body image changes among HSCT survivors through qualitative research. This approach could yield new and valuable insights, facilitating the development of targeted programs to address body image disturbances in HSCT survivors while enhancing overall survivorship care by integrating the body image dimension into holistic care practices. This research could also open new directions for future studies focused on supporting body image in this cohort.

However, qualitative evidence on body image changes in HSCT survivors remains limited and is typically addressed only as a minor aspect of their overall survivorship experiences.^9^^,^^16^^,^^18^, ^19^, ^20^, ^21^ No comprehensive qualitative studies have specifically targeted this research topic and explore it in depth. While qualitative evidence on body image changes in solid cancer survivors, such as those with breast or head and neck cancer,^22^^,^^23^ is available, these findings cannot be directly applied to HSCT survivors due to differences in treatment methods (localized treatments, typically associated with solid cancers, whereas HSCT involves systemic treatments for hematological malignancies) and the complex and lasting negative impacts of HSCT on the body and psyche.

Furthermore, body image experiences are closely related to local culture (e.g., a set of beliefs, values, traditions, ideologies, and social norms that characterize a group), and perceptions vary across different cultural perspectives (e.g., Western versus Asian cultures).^24^ For example, Western countries consistently revere the thin-ideal and fit-ideal as the dominant preferred body shape, emphasizing the malleability of the body's appearance and the belief that with sufficient effort, the ideal is attainable.^24^ By contrast, many Asian regions and countries, particularly China—one of the most populous countries in East Asia with a continuous history of around 5000 years—are deeply rooted in Confucianism.^24^ Key concepts, such as “relationship dominance” and “self and identity being embedded in relationships,” can influence how individuals perceive their bodies.^24^

A culturally and contextually grounded qualitative exploration is essential to gain a deeper understanding of the body image changes experienced by HSCT survivors. Findings derived from a targeted cultural framework could be valuable for developing feasible and acceptable interventions and guiding health care providers and caregivers in enhancing their support for HSCT survivors in addressing body image concerns. Therefore, this study aimed to explore the experiences of body image changes among HSCT survivors in China and examine how these changes influence their perception of self and body within the Chinese cultural context.

## Methods

### Research design

Descriptive qualitative research, grounded in the principles of naturalistic inquiry,^25^ was adopted as the overarching methodological approach to generate straightforward descriptions of the phenomenon in a manner that best aligns with the data.^26^ Notably, although description is central to this approach, low-inference interpretation is always present in the analysis and findings.^26^, ^27^, ^28^ Merleau-Ponty's phenomenology of embodiment, emphasizing the lived, embodied experience of the body and how individuals perceive and relate to their bodies in the world,^14^ serves as the theoretical lens for understanding these experiences in the current study.

This choice of methodology and theoretical lens is well-suited for addressing the research questions and aligns with the researchers’ worldviews, which embrace subjectivity and multiple realities (e.g., recognizing the subjective experience of the body), understanding phenomena in the natural context (e.g., interacting with and making sense of the world), and adopting a holistic perspective (e.g., emphasizing the inseparable connection between body and consciousness in shaping identity and existence). The Consolidated Criteria for Reporting Qualitative Research checklist was used to guide the reporting of this study (Supplementary File S1).^29^

### Recruitment and participants

Purposive sampling was utilized to identify potential information-rich HSCT survivors treated one tertiary-teaching hospital at Zhejiang Province, Southeast China. Maximum variation sampling was utilized for gender, age, marital status, educational level, disease type, and HSCT types.

The inclusion criteria involved the following: (1) a verified medical diagnosis of blood cancer, (2) ​≥ ​100 days post HSCT,^30^ (this decision was made in consideration of the elevated risks of acute GVHD and mortality within the first 100 days following HSCT, which could reduce the feasibility of the study), (3) no verified cancer recurrence, (4) aged at least 18 years old, (5) proficiency in understanding and speaking Chinese, and (6) willing to share experiences of body image changes. The exclusion criteria included the following: (1) being pregnant, (2) having a diagnosis of other health illnesses, which may influence a person's body image (e.g., endometriosis and eating disorders)^31^^,^^32^; and (3) being diagnosed with cognitive impairment, psychiatric disease, or severe physical impairment or inability to understand the study. A total of 23 HSCT survivors were invited. One became ineligible due to cancer relapses (*n* ​= ​1). Two declined to participate owing to a lack of interest (*n* ​= ​1) and time conflict (*n* ​= ​1).

### Data collection

Data were collected via semi-structured individual interviews from June 2021 to October 2024. An interview guide was created on the basis of literature involving body image changes in HSCT survivors,^18^, ^19^, ^20^, ^21^^,^^30^ other cancer survivors,^33^ and clinical experiences of HSCT nurses and hematologists (Table 1). The interview questions became more structured as the study progressed.

**Table 1:** Interview guide.

Interview questions
1. Please share with me how your body image has changed during survivorship after undergoing hematopoietic stem cell transplantation.
2. Please share your perceptions/feelings/thoughts when you notice changes after undergoing hematopoietic stem cell transplantation during survivorship.
3. Please share the key events related to your body image experiences after undergoing hematopoietic stem cell transplantation during survivorship.
4. Please share with me how these body image changes have impacted your life after undergoing hematopoietic stem cell transplantation during survivorship.
5. Please share with me anything you have learned through the process of experiencing changes in body image after undergoing hematopoietic stem cell transplantation during survivorship.

Interviews were conducted by JR, XL, or YQ, either face-to-face or online. JR, an experienced qualitative researcher who conducted over 100 successful qualitative interviews, provided ongoing training to XL and YQ, who are novice qualitative researchers. Before their interviews, JR explained the principles of descriptive qualitative research, the research questions, and the interview guide, and shared relevant materials on qualitative interview techniques. After each interview, XL and YQ engaged in self-reflection by reviewing their recordings and identifying strengths, areas for improvement, and any concerns. JR offered feedback on the basis of their reflections, with further guidance as needed. This iterative process helped XL and YQ progressively enhance their qualitative interviewing skills.

Interview settings were selected on the basis of the participant's preference, including physician's office, wards, homes of participants, restaurants, participant's workplace, head nurse's office, and WeChat (A Chinese mobile messaging application and social networking platform that that supports video communication). Probing questions were used to clarify participants' responses. For instance, when one participant expressed that she used to enjoy taking selfies but hardly took photos anymore post HSCT, XL inquired further: “Could you please share what has made you unwilling to take photos of yourself now?” Field notes regarding the interview setting and the nonverbal language and interactions between the interviewer and the interviewee were taken by the interviewer after each interview. All interviews were audio-recorded and transcribed verbatim. When the data collected were sufficient to develop rich, complex, and multifaceted findings about the patterns related to the studied phenomenon,^28^^,^^34^^,^^35^ also known as “data sufficiency,” data were no longer collected.

### Data analysis

Data were manually analyzed using the conventional content analysis.^36^ The analysis was conducted simultaneously with data collection by JR, who has rich experience in applying this method. First, JR read and reread the interview transcript several times to obtain an overall understanding of the experiences of body image changes in Chinese HSCT survivors. She then conducted line-by-line coding for the manuscript manually to identify coding units that captured key ideas about experiences of body image changes in HSCT survivors. Subsequently, the researcher compared these generated coding units and condensed coding units to a higher abstract level on the basis of shared characteristics. Lastly, some abstract coding units were extracted and categorized into subthemes and themes. Data analysis was conducted in Chinese, and subthemes, themes, and relevant quotes were translated into English for report writing by JR, who is bilingual in English and Chinese. The translated findings were reviewed by the research team, and disagreements were addressed by discussion.

### Rigor and trustworthiness

The rigor of this study was ensured through four key criteria: credibility, transferability, dependability, and confirmability.^25^^,^^37^ Credibility was established through prolonged engagement over the course of the study. All three interviewers, each with at least 3 years of experience as HSCT nurses, provided care to all participants prior to the interviews. This existing familiarity fostered trust, which was maintained throughout the interview process and facilitated a deeper understanding of participants’ perspectives.

Transferability was supported through comprehensive and detailed descriptions of the study context and sampling strategies. The research context, participant characteristics, methodology, sampling methods, and selection criteria were thoroughly and clearly articulated to enable readers to assess the applicability and relevance of the findings to other settings or situations and to determine whether the results may be transferable to similar populations or contexts beyond the original study.

Dependability was achieved through detailed methodological documentation and the use of audit trails. The research process was systematically recorded, and a transparent log of key decisions, including any changes made during data analysis, was maintained to enhance the study's transparency and trustworthiness.

Confirmability was ensured through reflexive journaling. All three interviewers actively maintained reflexive journals throughout the study. Prior to the interviews, thoughts and perceptions regarding the research topic were bracketed, and the reviewers reflected on how their roles (e.g., JR, XL, and YQ, each with experience as young female HSCT nurses, conducted the interviews) and any evolving thoughts or biases may influence data collection, analysis, and interpretation.

## Results

A total of 20 HSCT survivors participated in this study, with 18 attending the interviews face-to-face and two participating online. The average interview length was 82.65 min, ranging from 45 min to 123 min. All participants were Han Chinese, with a mean age of 39.2 years. Participants were coded as P1–P20. The socio-demographic and clinical characteristics of the participants are presented in Table 2.

**Table 2:** Study participants’ socio-demographic and clinical characteristics.

ID	Gender	Age (years)	Marital status	No. of children	Education level	Occupation	Disease type	Transplant type	Time since transplant (months)	GVHD during survivorship (involving organs)	Current general health status^a^
1	Male	51	Married	2	Primary or less	Self-employed businessman	Myelodysplastic syndrome	Allo-HSCT	19	Yes (skin)	Excellent
2	Male	47	Married	2	Secondary	Self-employed businessman	Acute myeloid leukemia	Allo-HSCT	27	Yes (skin and eyes)	Excellent
3	Female	59	Married	1	Secondary	Retired	Non-Hodgkin lymphoma	Auto-HSCT	7	NA	Excellent
4	Male	27	Married	1	Secondary	Freelancer	Acute myeloid leukemia	Allo-HSCT	19	Yes (liver, skin, eyes, and gastrointestinal tract)	Very bad
5	Male	32	Married	2	Secondary	Part-time online gaming jobs (homeworking)	Acute myeloid leukemia	Allo-HSCT	24	Yes (skin, lung, and eyes)	Poor
6	Female	36	Separated	1	Secondary	Unemployed and not looking for work currently	Acute lymphocytic leukemia	Allo-HSCT	46	Yes (skin, mouth)	Good
7	Male	28	Divorced	1	Secondary	Part-time network operation (homeworking)	Acute lymphocytic leukemia	Allo-HSCT	15	Yes (skin)	Good
8	Female	25	Single	NA	Tertiary or above	Unemployed and not looking for work currently	Acute lymphocytic leukemia	Allo-HSCT	8	Yes (skin)	Fair
9	Female	33	Married	2	Tertiary or above	Unemployed and not looking for work currently	Acute myeloid leukemia	Allo-HSCT	15	Yes (skin)	Good
10	Female	44	Married	1	Primary or less	Unemployed and not looking for work currently	Acute myeloid leukemia	Allo-HSCT	34	Yes (liver, mouth, and gastrointestinal tract)	Excellent
11	Female	18	Single	NA	Secondary	Full-time waitress at a hotpot restaurant	Acute lymphocytic leukemia	Allo-HSCT	32	Yes (mouth)	Very good
12	Male	43	Married	1	Tertiary or above	Full-time office clerk at a public institution (sick leave)	Non-Hodgkin lymphoma	Auto-HSCT	8	NA	Good
13	Female	22	Single	NA	Tertiary or above	Unemployed and not looking for work currently	Acute lymphocytic leukemia	Allo-HSCT	19	Yes (eyes)	Good
14	Male	35	Married	1	Tertiary or above	Full-time employee at a medical corporation	Non-Hodgkin lymphoma	Auto-HSCT	41	NA	Very good
15	Female	47	Married	1	Tertiary or above	Full-time primary school teacher (sick leave)	Acute lymphocytic leukemia	Allo-HSCT	20	Yes (skin)	Good
16	Female	49	Married	1	Secondary	Unemployed and not looking for work currently	Myelodysplastic syndrome	Allo-HSCT	6	Yes (bladder)	Good
17	Male	33	Married	1	Secondary	Full-time office clerk	Myelodysplastic syndrome	Allo-HSCT	35	Yes (liver and gastrointestinal tract)	Good
18	Female	56	Married	2	Primary or less	Unemployed and not looking for work currently	Non-Hodgkin lymphoma	Auto-HSCT	6	NA	Good
19	Female	54	Married	1	Secondary	Retired		Allo-HSCT	14	Yes (skin)	Good
20	Male	45	Married	1	Tertiary or above	Full-time office clerk (sick leave)	Non-Hodgkin lymphoma	Allo-HSCT	6	Yes (skin)	Good

Allo-HSCT, allogeneic hematopoietic stem cell transplantation; Auto-HSCT, autologous hematopoietic stem cell transplantation; GVHD, graft-versus-host-disease; NA, not applicable.

a Measured using a self-designed five-point Likert scale with the question: “How do you rate your current general health status?” (responses: excellent, good, fair, poor, very poor).

Three themes and 11 subthemes were identified as important aspects of experiences of body image changes in Chinese HSCT survivors: (1) I am an inferior person (a distinctly weird appearance, an impaired body function, and depending on external resources for survival); (2) I am a person struggling to reconcile with my changed body (struggling to conceal or disclose my changed body, negotiating the impact of my changed body on my work identity, struggling to understand and care for my changed body, and catching up in rumination over my changed body); and (3) I am a brand-new person (having escaped from the gate of hell, enhanced appearance, optimized personality, possessed sublimated emotions). Table 3 presents the themes, subthemes, and corresponding supporting quotes in English and Chinese.

**Table 3:** Themes, subthemes and corresponding supporting quotes of experiences of body image changes in Chinese hematopoietic stem cell transplantation survivors.

Themes	Subthemes	Quote exemplars	
		English language	Chinese language
I am an inferior person	A distinctly weird appearance	*“(After taking hormones), the hair around my mouth became particularly thick. It was fine when wearing a mask, but once I took it off, I looked like a man dressed as a woman—it looked so weird! … My face swelled up like a balloon. My abdomen also became very large, to the point where the buttons on my pants would pop off.”* (P8)	*“(吃激素)这(嘴边)一圈(胡子)特别浓,戴着口罩还好,一摘掉,跟男扮女装一样,好变态 … 脸肿的跟气球一样 … 肚子会变得很大,(胖到)把裤子的扣子都崩飞了”* (P8)
		*“Previously, I had long hair, but now it is short. My hair sticks up completely, it's curly and soft, which looks quite strange.”* (P18)	*“我以前是长头发,现在短头发。头发全都翘起来,卷卷,软软的,怪怪的”* (P18)
		*“The hair that's grown out is soft, fluffy, and white—grayish white, like that of an old woman. It's so ugly!”* (P16)	*“长出来的头发,又软、又绵、又白,灰白的那种,跟老太婆一样。难看死了!”* (P16)
		*“At that time (early post-transplantation), I was just like a black fish.”* (P1)	*“那时(移植后早期) (我)像一条黑鱼一样”* (P1)
		*“When I was back home (after HSCT), my face was dark, just like a black-boned chicken.”* (P2)	*“(移植后)回来的时候,我的脸黑黑的,就跟乌骨鸡一样”* (P2)
		*“(In my residential community) someone would avoid me but greeted my husband and then pointed at me, asking, ‘How is your mother doing?’ … When traveling by high-speed train, the attendants said to my husband, ‘Support your mother properly.’”* (P15)	*“(小区有人)看到我就走了,但跟我老公打招呼,然后指着我说,‘你老妈(情况)怎么样?’ … 那时, 坐高铁, 服务员也是,(对我老公说),‘把你妈扶好’”* (P15)
		*“I used to weigh 168 jin (*84 kg*) … I have never been this thin before! … I am 1.68 ​m tall and weigh only 110 jin (*55 kg*). I can feel my ribs clearly, one by one!”* (P17)	*“我以前168斤 … 我从来没这么瘦过!1米68,就110斤。肋骨都能一根一根的,摸得很清楚”* (P17)
		*“A young person who doesn't look young, but like a fifty- or sixty-year-old man! Who can accept that?”* (P5)	*“一个年轻人不像个年轻人,跟个五、六十岁的老头子一样! 谁能接受呢?”* (P5)
		*“My skin has become so dark, with patches all over my body. So unattractive!”* (P6)	*“皮肤变得黑,身上都一块块的(斑)。很难看啊!”* (P6)
	An impaired body function	*“My vision was blurry … (Doctor said), my eyelashes were growing inwards and poking my eyeball.”* (P10)	*“眼睛看东西不清楚 … (医生说),眼睫毛长反了,都戳到眼珠子”* (P10)
		*“After the HSCT, I couldn't see anything clearly when I looked at my phone.”* (P19)	*“移植做过,眼睛看手机,根本看不清楚”* (P19)
		*“Oral graft-versus-host disease was so severe … all (teeth) were decayed, like half-rotten, that kind of decay.”* (P11)	*“口腔排异的太厉害了 … 全部(牙)都是烂的,一半、一半的,那种烂”* (P11)
		*“My memory has declined … it's kind of like a fish's memory now.”* (P13)	*“记忆力退化 … 有点跟那种鱼的记忆一样了”* (P13)
		*“At that time, my muscles had atrophied, and I couldn't squat down to use the bathroom.”* (P11)	*“我那时肌肉萎缩了,上卫生间蹲不下去”* (P11)
		*“(After the transplantation), it's harder to manage than after chemotherapy. Just a breeze, and you might not make it (getting infected). I'm really fragile now.”* (P15)	*“(移植后)比以前化疗后,难伺候。风一吹,你可能就不行(感染)。现在真的很脆弱”* (P15)
		*“People like us, who came out of the transplant isolation room, really need to be specially protected … I told them (friends), ‘I am a panda, don't come see me.’”* (P20)	*“像我们这种仓里出来的人,真的要特别保护 … 我跟他们 (朋友)说,‘我是熊猫,不要来看我’”* (P19)
		*“When climbing stairs, my feet had no strength.”* (P20)	*“爬楼梯,两个脚没力气”* (P20)
	Depending on external resources for survival	*“So many pills. Sometimes the medication makes you want to vomit, especially the large pills.”* (P8)	*“好多药(要吃)。有时,吃药吃的都想吐,特别是那种大颗的”* (P8)
		*“Sometimes, just thinking about having to take so many pills after a meal, a whole handful—feels really hard to bear! Every meal comes with a handful of pills!”* (P17)	*“有时,想到每顿饭后要吃这么多药,一把一把的,太痛苦了。每顿饭都要吃一把药!”* (P17)
		*“It's lucky that we have health insurance, so it didn't cost that much.”* (P9)	*“还好有医保,有保险,花不了那么多钱”* (P9)
		*“I felt like a useless person, unable to do anything except taking medication!”* (P8)	*“我就是个没用的人,除了吃药,什么也做不了!”* (P8)
I am a person struggling to reconcile with my changed body	Struggling to conceal or disclose my changed body	*“When colleagues asked, ‘Have your period come? Why haven't I seen you get your period?’ Sometimes, I responded that it had happened, but you just didn't notice. But it was mentally stressful, fearing that they would discover that (I no longer have my period post-HSCT).”* (P11)	*“同事问我,‘你的月经来了吗?我怎么没看见你来月经?’有时,只能说,已经来过了,只不过你们没看见。但是心理很有压力,生怕他们发现 (移植后,我没有月经的情况)”* (P11)
		*“It's conflicting. Sometimes, I wanted to disclose to others. I was indeed weak and needed care. But at the same time, I wanted to be treated as a normal person and avoided disclosure.”* (P7)	*“很奇怪。有时候,因为确实是弱,需要被大家照顾,(想告知他们)。但是,好像,又确实希望被大家当成一个正常人对待,(而避免告知)”* (P7)
	Negotiating the impact of my changed body on my work identity	*“Think about it, for a family like ours, how could I not go to work? I still have two elderly people who need support, one mother-in-law and one mother.”* (P18)	*“你想想,我们这么一家人,我怎么能不去上班?我还有两个老的要养,一个婆婆,一个妈妈”* (P18)
		*“Basically, I think about business every day. Some people say, (suffered from cancer and transplantation) you shouldn't be focused on doing business. But if you don't do business, when the money runs out, how will you pay for hospitalization?”* (P4)	*“基本上,每天想着生意。有些人说,(经历过患癌与移植) 不应该动心思去做生意。但你不做生意,你没钱了,拿什么来住院呢?”* (P4)
		*“My boss and colleagues asked, ‘why don't you get the vaccine? … I was stalling. I was afraid to get the shot (due to potential side effects). Meanwhile, I feared losing the job.”* (P11)	*“领导和同事问道,‘为什么你不打(新冠疫苗)?’ … 先拖着。我不敢打(万一打了有什么事情)。但我也怕(因这个原因)丢工作”* (P11)
	Struggling to understand and care for my changed body	*“Another survivor told me that the relapse was due to exercise, making me quite nervous. Now I don't even know if I can exercise or not.”* (P13)	*“另外一个(移植后)病人告诉我,他运动复发的。弄得我有点慌兮兮的,都不知道能不能运动了”* (P13)
		*“The TCM physician said that you need to avoid certain triggering foods like chicken. But the doctor here (HSCT doctor) told me no need to avoid any food … I was quite confused! (Finally) I stopped eating the chicken I bought and only ate the ones we raised ourselves.”* (P10)	*“中医医生让我忌口,避免发物,如鸡。但这边的医生(移植医生)告诉我不用忌口 … 弄不清楚。(最终)我买的鸡都不吃,自己家里养的那个(鸡)吃”* (P10)
		*“It's a conflicting situation when it comes to body care. On one hand, you need a strong immune system, but on the other hand, you can't let it be too strong.”* (P16)	*“调理(身体),矛盾的状态。因为你一方面,抵抗力要好,一方面,又不能让你(抵抗力)太好”* (P16)
	Catching up in rumination over my changed body	*“I could not tolerate any pain in my body. If I felt any pain, I started thinking, ‘What situation do I have now? I became very anxious and full of self-doubt!’”* (P13)	*“我身上不能有哪一点痛。这一痛,我就觉得,‘完了,我生了什么病?’ 就是很焦虑,自我怀疑”* (P13)
		*“I kept wondering when this (severe GVHD) would end. How long would this condition last? When could I recover a bit better? I just couldn't get better; the rejection was overwhelming!”* (P5)	*“总是不停想,这种(严重排异)状态,什么时候是个头? 什么时候可以恢复得好一点? 一直恢复不过来,排异特别多!”* (P5)
		*“In a patient support group, I saw other patients mention where they felt unwell. I got scared (that I might be the same). Really scared! I muted the group and stopped checking it.”* (P7)	*“有个病友群,我看到,他们(病友)会说一下,哪里不舒服。我害怕(自己也那样)。真的害怕。我把群屏蔽掉了,不去看”* (P7)
I am a brand-new person	Having escaped from the gate of hell	*“One checkpoint after another. Just like those TV shows where you passed through levels. I have passed one checkpoint after another.”* (P10)	*“一道关、一道关过。就像电视里面,闯关一样。我已经一道、一道闯过去了”* (P10)
		*“It was truly unbearable! A kind of torment that no ordinary person could endure … So unforgettable! I even wrote my will … Look back, I have really been at death's door several times.”* (P4)	*“真的是太不容易了! 真的是常人难以忍受的煎熬 … 真是太刻骨铭心了。我遗嘱都写好了 … 这一路走过来,真的是,走了好几回鬼门关了”* (P4)
		“*So many people died in the hospital post transplantation, or they just gave up … It really feels like I have been reborn! I have become a new person now.”* (P1)	*“医院(移植后)死掉的人太多了,要不就是放弃了 … 我重生了! 重新做人了,现在”* (P1)
		*“It really feels like a rebirth. We are the lucky ones in the disaster. I heard that many people who underwent the transplantation around the same time as I have passed away.”* (P13)	*“真的算重生了。我们就是灾难里的幸运者。我听见,跟我差不多一起(时间)移植的,很多人都走(死)了”* (P13)
	Enhanced appearance	*“When I came back, I weighed only 108 jin (*54 kg*), now I'm over 140 jin (*70 kg*).”* (P2)	*“回来的时候,只有108斤,现在140多斤了”* (P2)
		*“My weight dropped. I was quite happy about it. Being slim looks good!”* (P11)	*“体重下降了,我还挺高兴的。瘦嘛,好看呀!”* (P11)
		*“My skin is completely different now; it has become white and much fairer.”* (P1)	*“皮肤,现在都不一样了,变得很白净了”* (P1)
	Optimized personality	*“I was self-centered before … If I went out, I would go wherever I thought that it was fun not considering the feelings of those going with me. But now I think about their thoughts.”* (P7)	*“以前,以自我为中心 … 如果出去玩,我感觉哪里好玩就要去哪里,不会考虑一起去人的感受。但是现在,我会考虑他们的想法”* (P7)
		*“I would always insist on making my point, reasoning, and sticking to principles. But after going through this serious illness and treatment, it taught me something. Some things just weren't worth it, and I don't bother with them anymore.”* (P15)	*“我以前总是坚持己见,讲道理,讲原则。但经过了这场大病和治疗后,也教会了我一些东西。有些东西根本不值得,我也就不再计较这些”* (P15)
		*“After going through so many storms and enduring so much hardship, there's no other suffering that can surpass it (transplantation). I've made it through!”* (P2)	*“经过了这么多风雨,吃了这么多苦头。已经没有其他苦头可以超过它(移植)了,都熬过去了!”* (P2)
	Possessed sublimated emotions	*“My wife has been with me through the illness. She is pregnant now, and that makes me very happy!”* (14)	*“从疾病到现在,我老婆都在(我身边)。现在她怀孕了,挺开心的!”* (P14)
		*“Honestly, without my husband, I definitely couldn't have made it through my illness.”* (P9)	*“真的,我生病没有我老公,绝对挺不过来”* (P9)
		*“He (my husband) always made me happy. Whatever I wanted to eat, he cooked delicious food for me.”* (P3)	*“他(丈夫)总让我开心。我想吃什么,他都会烧好吃的给我吃”* (P3)
		*“My daughter was very dutiful and considerate; she helped me organize my medication (after the transplant).”* (P10)	*“女儿,蛮孝顺的,蛮懂事的,帮我把(移植后口服)药都摆好”* (P10)
		*“My boss was quite caring and considerate. He had visited me at home several times and tried his best to apply for any available benefits for me.”* (P12)	*“我单位领导还是比较热心的,人性化的。来家里也看过我好几次。能申请的补助,也都尽量给我申请过了”* (P12)

### Theme 1: I am an inferior person

Theme 1 reflects that HSCT survivors described themselves as inferior individuals. This negative self-evaluation is primarily related to their weird appearance, impaired normal physical functions, and reliance on external resources to survive. These feelings are especially common during the early survivorship phase or during episodes of severe GVHD, when survivors are deeply preoccupied with their changed body image and may exhibit aversion, rejection, or even denial toward these transformations.

#### A distinctly weird appearance

Most HSCT survivors experienced unusual appearances at various stages of survivorship. These appearances included “moon face,” “facial and body hair growth,” or “increased central adiposity”; “baldness” or “fine, short, soft and curly new hair”; “gray and dull,” “like a black fish” or “like a black-boned chicken”; “look like an old man” (young man actually), “mistaken for my husband's mother”; “like a corpse,” “nothing but skin and bones”. As one participant said, “I used to weigh 168 jin (84 kg) … I have never been this thin before! … I am 1.68 ​m tall and weigh only 110 jin (55 kg). I can feel my ribs clearly, one by one!” (P17) Another participant said, “A young person who doesn't look young, but like a 50- or sixty-year-old man! Who can accept that?” (P5) The generation of these appearances were related to disease treatments (e.g., chemotherapy, steroids, HSCT, and immunosuppressants like cyclosporine and tacrolimus), post-transplant hair regrowth, varying degrees of GVHD affecting different organs, and menopause or endocrine disorders.

Overall, identifying with the experience of “looking sick” led participants to feel self-conscious, feel low in mood, and even lose confidence. Descriptions, such as “ugly,” “unattractive,” and “like a monster,” reflected their deep aversion to these unusual appearances. “The hair that's grown out is soft, fluffy, and white—grayish white, like that of an old woman. It's so ugly!” (P16) “My skin has become so dark, with patches all over my body. So unattractive!” (P6).

#### An impaired body function

Impaired body function may be related to disease (e.g., brain lymphoma), pre-HSCT conditioning, the side effects of HSCT, and GVHD. “Seeing like someone with severe myopia,” “like a blind person,” “blurry vision,” and “upturned eyelashes” reflected damage to eye function. “Rotten teeth,” “peeling lips,” and “altered taste” indicated damage to oral function. “Like a fish's memory,” “hand tremors,” and “muscle atrophy” reflected impaired neurological and cognitive functions. “Amenorrhea” pointed to reproductive and fertility function impairment. Descriptions, such as “weak and delicate,” “very difficult to care for,” “like a giant panda,” and “treated like a royal,” revealed impaired immune function. “Fatigue,” “sore legs,” and “leg pain” represented multifunctional impairments, including impaired musculoskeletal function, immune function, systemic fatigue, and nerve function. As participants mentioned, “After the HSCT, I couldn't see anything clearly when I looked at my phone.” (P19) “When climbing stairs, my feet had no strength.” (P20).

#### Depending on external resources for survival

Relying on external resources for survival is crucial for HSCT survivors to maintain existence. One aspect is “centering medical resources.” It included medication after HSCT, medical institutions that performed the transplant and/or treated patients post-HSCT, and medical insurance. Most HSCT survivors needed to take a huge amount of medicine daily to prevent infections and rejections. This situation made participants feel disgusted and unacceptable, and some even metaphorized themselves as “medication containers.” “So many pills. Sometimes the medication makes you want to vomit, especially the large pills.” (P8) and “Sometimes, just thinking about having to take so many pills after a meal, a whole handful—feels really hard to bear! Every meal comes with a handful of pills!” (P17) Another reflection is “relying on others” for survival. When the reliance was intense and recurring and its duration was difficult to foresee, survivors evaluated themselves as “a useless person.” Meanwhile, feelings of “worthlessness” and “guilt” generated. “I felt like a useless person, unable to do anything except taking medication!” (P8).

### Theme 2: I am a person struggling to reconcile with my changed body

Theme 2 captures the experiences of HSCT survivors who struggle to reconcile with their changed bodies during the survivorship period. Over time, as survivors came to recognize that their changed body image was an enduring reality, they began to understand that acceptance was the only way to move forward. From this point, they started to learn and practice how to live with their changed bodies amid ongoing challenges. Four key areas were identified: struggling to conceal or disclose my changed body, negotiating the impact of my changed body on my work identity, struggling to understand and care for my changed body, and catching up in rumination over my changed body.

#### Struggling to conceal or disclose my changed body

Disclosing one's changed body is a double-edged sword. It can bring benefits, such as receiving care, attention, and financial support, to survivors. However, it can also result in being overly cared for as a cancer survivor, experiencing indifference, isolation, or even negative judgment. In most cases, survivors choose to keep their condition private after assessing the potential pros and cons. However, in some instances, survivors were intensely disturbed by an inner struggle over whether to disclose their altered body, such as when confronting with inquiries (especially when what they attempted to conceal were inadvertently discovered). “When colleagues asked, ‘Have your period come? why haven't I seen you get your period?’ Sometimes, I responded that it had happened, but you just didn't notice. But it was mentally stressful, fearing that they would discover that (I no longer have my period post-HSCT).” (P11) “It's conflicting. Sometimes, I wanted to disclose to others. I was indeed weak and needed care. But at the same time, I wanted to be treated as a normal person and avoided disclosure.” (P7).

#### Negotiating the impact of my changed body on my work identity

Most HSCT survivors, especially those who received allo-HSCT, chose to stay at home during the recovery period and waited 3–5 years before attempting to return to the workforce. However, when survivors and their families were in significant economic need, the question of whether to return to work sooner became their main struggle. Despite being in the early stage of survivorship and experiencing physical changes like GVHD, some survivors felt compelled to return to work, often at the cost of their health. “Think about it, for a family like ours, how could I not go to work? I still have two elderly people who need support, one mother-in-law and one mother.” (P18).

Even after returning to work, survivors continued to struggle with the impact of their changed bodies, such as physical discomfort, or needing to manage specific health precautions like vaccination. These challenges not only affected their ability to meet job demands but also influenced how they were perceived in the workplace. For instance, one participant was caught in a dilemma between receiving the coronavirus disease 2019 vaccine, as required by their workplace but with potential side effects, and avoiding the vaccine, which carried a high risk of being fired. “My boss and colleagues asked, ‘why didn't you get the vaccine?’ … I was stalling. I was afraid to get the shot (due to potential side effects). Meanwhile, I feared losing the job.” (P11).

#### Struggling to understand and care for my changed body

Due to the lack of clear guidance on maintaining a healthy lifestyle post-HSCT, survivors often found it challenging to adjust to their changed bodies and make informed decisions about physical activity and diet. Many survivors were conflicted about questions like “Can I exercise?” “Is there a difference between types of exercise?” “What types of exercise are suitable for my body?” and “What foods can I eat?” and “What food should I avoid?” The uncertainty surrounding their altered bodies often led to anxiety, with some survivors even deciding to stop exercising altogether due to the fear of worsening their condition. “Another survivor told me that the relapse was due to exercise, making me quite nervous. Now I don't even know if I can exercise or not.” (P13).

Further complicating their body care was the inconsistency between Western medical advice and traditional Chinese medicine (TCM) recommendations. Western physicians generally advised no dietary restrictions, whereas TCM practitioners emphasized avoiding certain foods, such as chicken, leading to confusion and stress over what to eat. As one participant recalled, “The TCM physician said that you need to avoid certain triggering foods like chicken. But the physician (HSCT physician) here told me no need to avoid any food … I was quite confused! (Finally) I stopped eating the chicken I bought and only ate the ones we raised ourselves.” (P10).

The conflicting health guidance not only made it difficult for survivors to care for their changed bodies but also intensified their emotional struggle with adapting to their new physical selves. The uncertainty about what was “right” for their bodies added to their sense of vulnerability and confusion about how to live in a body that no longer felt like the one they once knew.

#### Catching up in rumination over my changed body

The uncertainty surrounding physical changes to their bodies, such as the onset or worsening of GVHD, led survivors to ruminate intensely on their body. Any slight discomfort in their bodies can trigger overwhelming thoughts. Survivors became mentally drained and anxious whenever they experienced physical discomfort. “I could not tolerate any pain in my body. If I felt any pain, I started thinking, ‘What situation do I have now? I became very anxious and full of self-doubt!’” (P13) Persistent GVHD symptoms with no signs of improvement intensified their fears, leading to negative emotions and heightened concerns about their appearance. “I kept wondering when this (severe GVHD) would end. How long would this condition last? When could I recover a bit better? I just couldn't get better; the rejection was overwhelming!” (P5).

Survivors also found that discussions in patient support groups or social media about others’ worsening body conditions, such as changes in physical appearance or health deterioration, made them more fearful. They internalized these negative experiences, leading to even greater rumination about their own altered bodies. One survivor shared, “In a patient support group, I saw other patients mention where they felt unwell. I got scared (that I might be the same). Really scared! I muted the group and stopped checking it.” (P7).

### Theme 3: I am a brand-new person

Theme 3 reflects HSCT survivors' perception during the survivorship period of becoming completely new individuals. This transformation is marked not only by regaining life but also by improvements in appearance, personality, and emotional connections. It shows not only the survivors’ acceptance of their changed body image but also their successful integration of this altered image into daily life, indicating a sense of coexistence with the transformed self. This theme is commonly observed among survivors who have reached a relatively stable state after enduring body image changes associated with adversity during their survivorship journey.

#### Having escaped from the gate of hell

The phrase “the gate of hell” vividly captures situations of extreme danger or life-or-death moments. Many survivors have expressed that they have faced several such moments since their cancer diagnosis. They likened these experiences to passing through multiple life-or-death checkpoints. “One checkpoint after another. Just like those TV shows where you passed through levels. I have passed one checkpoint after another.” (P10) These critical moments may be related to cancer diagnosis, HSCT treatment, and GVHD. Gastrointestinal GVHD leading to intestinal bleeding is an example. “It was truly unbearable! A kind of torment that no ordinary person could endure … So unforgettable! I even wrote my will … Look back, I have really been at death's door several times.” (P4).

Escaping these life-threatening situations was no easy feat. When survivors successfully pulled through, they described themselves as “reborn,” “gaining a second life,” “becoming a new person,” or “kicked back from the underworld.” A sense of fortunateness generated at the same time. “So many people died in the hospital post transplantation, or they just gave up … It really feels like I have been reborn! I have become a new person now.” (P1).

#### Enhanced appearance

As the post-transplant period lengthened, some survivors noticed improvements in their appearance. A male survivor mentioned that he gained weight and improved his physique. “When I came back, I weighed only 108 jin (54 kg), now I'm over 140 jin (70 kg).” (P2) Some survivors who were overweight before illness found that their weight was unconsciously brought under control during survivorship. They were relatively satisfied with their slimmer physique. “My weight dropped. I was quite happy about it. Being slim looks good!” (P11) Besides, some participants expressed that their skin improved during survivorship, becoming “fairer” than before illness. “My skin is completely different now; it has become white and much fairer.” (P1).

#### Optimized personality

After experiencing the challenges of diagnosis, HSCT, and others that occurred in the survivorship, some survivors expressed that their personalities improved. Some became more empathetic and could put themselves in others' shoes. “I was self-centered before … If I went out, I would go wherever I thought that it was fun not considering the feelings of those going with me. But now I think about their thoughts.” (P7) Some survivors became more open-minded. Their temperaments became gentler, and they rarely quarreled over trivial matters with others as they used to. “I would always insist on making my point, reasoning, and sticking to principles. But after going through this serious illness and treatment, it taught me something. Some things just weren't worth it, and I don't bother with them anymore.” (P15) Some survivors realized that their psychological resilience strengthened, and they became more determined. “After going through so many storms and enduring so much hardship, there's no other suffering that can surpass it (transplantation). I've made it through!” (P2).

#### Possessed sublimated emotions

When survivors faced difficulties since cancer diagnosis, the sincere love and support from others made them feel cared for and helped them identify who truly loved them. These experiences inadvertently deepen the survivors' trust in others and elevate their relationships. This phenomenon occurs among family members. The unwavering support, patience, and careful care from a partner can be especially comforting for survivors. “My wife has been with me through the illness. She is pregnant now, and that makes me very happy!” (14) “Honestly, without my husband, I definitely couldn't have made it through my illness.” (P9) “He (my husband) always made me happy. Whatever I wanted to eat, he cooked delicious food for me.” (P3) The thoughtful care of children also provided great comfort to survivors. “My daughter was very dutiful and considerate; she helped me organize my medication (after the transplant).” (P10) Support also comes from relatives and friends and can be found in the workplace. “My boss was quite caring and considerate. He had visited me at home several times and tried his best to apply for any available benefits for me.” (P12).

## Discussion

To the best of our knowledge, this qualitative study is the first to explore the experiences of body image changes in hematological cancer survivors, especially targeting those who have undergone HSCT. Findings not only provide context-specific information for developing future interventions to address negative body image but also support the development or refinement of a holistic survivor-centered, need-based survivorship care models and plans.

Body image is not a simple concept that ranges solely from negative to positive; rather, it is a multidimensional construct.^38^ This complexity is also evident in the body image experiences of Chinese HSCT survivors. Three key themes were identified, presenting a three-phase, temporal, and dynamic psychological acceptance process of altered body images among Chinese HSCT survivors throughout their survivorship journey. A development trajectory was revealed, evolving from “feelings of inferiority” and “aversion, rejection, or even denial toward bodily transformations,” to “reconciliation with the changed body,” and ultimately, to “achieving a sense of renewal and coexistence with the changed body.” It should be noted that the relationships among these phases are not always linear but can be iterative and may be influenced by various factors such as psychological inflexibility and the intensity of symptoms during survivorship. For example, a survivor in phase 1 may be more likely to progress to phase 2 earlier due to a higher level of psychological flexibility. Moreover, a survivor in phase 3 may return to phase 2 or even phase 1 following the sudden onset of GVHD during survivorship, with those experiencing more severe symptoms being more likely to return to phase 1. The psychological acceptance process appears to be more fluctuating, challenging, and prolonged compared to other chronic illnesses (e.g., breast cancer, spinal cord injury, and inflammatory bowel disease).^39^, ^40^, ^41^ Further studies are required to examine the psychological acceptance process of body image changes among HSCT survivors and the corresponding influencing factors.

The embodied experience integrates having a body and being a body, forming a foundational basis for the development of self-identity—a broader, more holistic concept that refers to the unique set of beliefs, values, and characteristics defining an individual.^42^^,^^43^ Through bodily experience (e.g., the body as felt or sensed), identity formation occurs.^43^^,^^44^ Among HSCT survivors, significant changes in body image can be accompanied by corresponding transformations in identity. Findings from the current study—such as bodily experiences as “a distinctly weird appearance” and “an impaired body function” leading to the perceiving themselves as “an inferior person”—support a potential link between altered body image and shifts in self-identity in this population. It is noteworthy that changes in body image do not necessarily lead to changes in self-identity. Future research is needed to further investigate the nature of this relationship (e.g., whether it is unidirectional or reciprocal or unidirectional but interact in a dynamic reciprocal fashion to form a coherent and clear sense of self)^44^ and to explore the underlying mechanisms (e.g., informational processes, neurophysiological pathways, and other influential factors).

Theme 1 “I am an inferior person” manifested the obvious negative body image in Chinese HSCT survivors and indicated the low self-esteem in this population. This finding is consistent with a previous cross-sectional survey, which stated that over 70% of patients that underwent HSCT felt less physically and sexually attractive.^15^ The present study vividly described the details of those negative body images throughout survivorship trajectory. Neglected negative body image in HSCT survivors, especially those who received allo-HSCT, appears more severe and shocking than in cancer survivors with better-studied body image issues, such as those with hand and neck cancer (e.g., weight loss, reconstructive surgery)^22^ or breast cancer (e.g., hair loss, impact of surgery).^45^ Thus, the negative health outcomes induced by negative body image (e.g., depression, anxiety, social isolation, poor quality of life) might be more common among HSCT survivors,^15^ although this assumption still requires more investigations to confirm.

Resembling with a former meta-synthesis represented the subtheme as “centered around hospital.”^9^ The current study not only indicated the dependence of hospitals but also pointed out the deep dependence on other medical resources like medications. Adhering to the prescribed medications can ensure the best outcomes and prevent complications of patients undergoing HSCT.^46^ However, some participants expressed their deep loathing for this tied identity as “medication containers” due to being heavily dependent on the complex medication regimen daily. This phenomenon may result in discontinuing practicing medication, contributing to the current high non-medication adherence rate (51%) of patients undergoing HSCT.^46^

Theme 2 presented the struggles of identity and body images penetrating different angles of a Chinese HSCT survivor's daily life and revealed the internal conflicts of different identities occurring under different circumstances. Similar findings were reported in HSCT survivors from the Netherlands.^21^ In the context of cancer survivorship, the phenomenon of a survivor owning various identities (e.g., the old, the precancer self, the patient self, the new, post-cancer self, and the GVHD self) and negotiating with different identities is not uncommon. Interactions with different identities can create challenges and result in a life full of uncertainty and distress.^47^ A notable detail that within the Chinese cultural context, these challenges and struggles induced during identity negotiation among HSCT survivors may be more severe while having some traits, which can be understood from the two aspects below.

The first aspect is related to Confucianism, with collectivism being one of its core manifestations. Unlike individualism—which is often observed in Western countries and emphasizes autonomy, independence from in-groups, prioritization of personal goals over collective goals, and behavior guided mainly by personal attitudes^48^^,^^49^—collectivism is characterized by the interdependence of individuals within in-groups, the prioritization of group goals over personal ones, behavioral regulation according to group norms, and a communal orientation.^48^, ^49^, ^50^ In East Asian collectivist cultures influenced by Confucianism, such as China, maintaining social harmony and relational obligations is considered essential.^50^ Individuals are expected to demonstrate behavioral consistency, obey collective expectations, contribute beyond their individual roles, and even sacrifice personal needs for the welfare of the group to achieve group cohesion and societal harmony.^48^^,^^49^ These deeply rooted cultural values help explain why Chinese HSCT survivors may struggle with the decision to disclose or conceal their changed body during survivorship. Actions that deviate from collective norms—such as choosing not to receive the coronavirus disease 2019 vaccine when the HSCT survivor (P11)'s boss and colleagues have already done so—may not only be perceived as personal choices but could also be viewed as behaviors that disrupt the harmony within the workplace. Such deviations can place survivors in challenging situations, potentially leading to inner conflict, emotional struggle, social isolation, or perceived judgement from the workplace community.

The central tenet of Confucianism is the pursuit of social harmony, with family harmony serving as its foundational building block. In the Chinese context, family harmony is closely tied to the principle of filial piety, which emphasizes respect, obedience, and care toward one's parents and elder family members.^24^^,^^51^ Under this deeply rooted cultural value, fulfilling familial duties is seen as a moral obligation rather than a personal choice. As a result, some Chinese HSCT survivors, deeply influenced by the cultural expectation of filial piety, feel compelled to return to the workforce prematurely to earn income and to care for elderly family members, even when doing so may conflict with their physical recovery or psychological well-being.

The second aspect relates to the distinctive features of the medical system in China, where TCM and Western medicine are widely practiced and integrated into the national insurance system. However, individuals may struggle to understand and care for their changed bodies after transplantation due to the lack of specific lifestyle guidelines for HSCT survivors. Receiving differing recommendations from practitioners of these two medical systems further adds to the confusion among Chinese HSCT survivors. More qualitative research is needed to explore the facilitators and barriers to adopting and maintaining a healthy lifestyle after HSCT. Evidence-based lifestyle guidelines should be developed through clinical trials and refined via expert consensus such as through a Delphi study.^52^

Theme 3: “I am a brand-new person” represents the consequence of accepting altered body image and reflects the emergence of a new identity during survivorship. Similar findings, such as “enhanced personal growth,” have been reported in a previous study.^9^ This theme echoes a concept that remains underexplored among cancer survivors—positive body image. Positive body image is a holistic and multifaceted construct, comprising six key traits: body acceptance or love, body appreciation, inner positivity, conceptualizing beauty broadly, adaptive investment in appearance, and interpreting information in a body-protective manner.^53^ Subtheme 3.2, “enhanced appearance,” directly aligns with the notion of body appreciation. Research has shown that positive body image is malleable and protective,^53^ and it is positively associated with a range of psychological well-being indicators.^54^

The development of positive body image is inherently cultural and context specific. The perception expressed in the subtheme “having escaped from the gate of hell” reflects elements of Taoism and folk religions, in which survivors expressed gratitude and perceived their relapse- and GVHD-free or mild-GVHD lives as a spiritual rebirth or a second chance at life. The subtheme “optimized personality,” particularly the strengthening of psychological resilience, resonates with the Confucian ideal of self-cultivation. It reflects the belief that personal transformation and transcendence can be achieved through endurance and perseverance in suffering and daily life.^24^ Meanwhile, the subtheme “possessing sublimated emotions” highlights the profound role of interpersonal relationships in the lives of Chinese HSCT survivors—particularly familial relationships. It reflects the enduring importance of family bonds in the survivorship phase, where emotional support, a sense of responsibility, and relational harmony are deeply valued. Such emphasis on relationships strongly echoes Confucian values, which emphasize social connections and place family at the core of social and moral life.^24^^,^^50^ Future research should further explore the cultural characteristics of positive body image in HSCT survivors, examine the influential factors behind its development, and validate the culturally grounded patterns initially identified in this study. Ultimately, this may support the construction of a holistic, culturally informed model of positive body image tailored to this population.

### Limitations

This study has some limitations. First, participants were selected from clinical records of a single tertiary hospital in Zhejiang Province, Southeast China, which may limit the geographic and socioeconomic diversity of the sample. Additionally, the maximum survivorship duration was 46 months, which may not fully capture the experiences of body image changes in long-term survivors. Future studies should consider multicenter recruitment and include participants with longer survivorship durations to enhance the generalizability and depth of the findings. Second, limited findings regarding sexual dysfunction and related impacts were obtained, although sexual dysfunction is common in males and females after HSCT.^55^ Third, the present study captures participants’ body image experiences at a single point in time. Future research should consider longitudinal designs—qualitative, quantitative, or mixed-methods—to explore the dynamic changes in the three identified levels of body image experiences over time, thereby deepening the understanding of these dynamics in HSCT survivors. Moreover, although the relationships among the three themes have been identified as not always linear but iterative, further in-depth exploration is needed to better understand the relationships and interactions between these themes. Lastly, the interview questions were not piloted, which may have led participants toward specific reflections and overlooked those who did not frame their experiences in terms of body image, though the risk is minimal. Future studies should consider conducting pilot qualitative interviews.

### Implications for clinical practice and research

Health care providers, especially HSCT nurses, primary care nurses, and community nurses, play a central role in the care of HSCT survivors during their survivorship. These providers should be aware that HSCT survivors may experience varying degrees of body image distress throughout survivorship. The prevalence of body image distress and related psychosocial risk factors in HSCT survivors should be investigated to better understand negative body image and more accurately identify the affected population. Regarding the negative emotions and struggles that arise when negotiating identities during survivorship, HSCT physicians, nurses, and family caregivers should continuously monitor survivors’ psychological well-being. A proposed intervention framework, such as psychosocial support models, is essential to provide actionable guidance. With this framework in place, appropriate psychological interventions should be developed and applied timely to reduce body image distress.^56^^,^^57^ Additionally, further research is needed to explore how social support and identity are communicated among HSCT survivors and their social networks to improve the management of identity negotiations.

Moreover, the limited discussion of sexual dysfunction among HSCT survivors raises important questions about cultural sensitivity. Although participants in this study did not explicitly mention concerns related to sexual dysfunction, their silence may reflect cultural taboos rather than the absence of such issues. This phenomenon is likely related to the conservative cultural context in China, where sexuality is often regarded as a sensitive, private, shameful, and even taboo topic.^58^ The fear of “losing face” (丢脸, *“diu lian”*), a concept rooted in Confucianism and deeply embedded in Chinese society, goes beyond mere embarrassment, shame, and humiliation to include the loss of respect, reputation, or social status.^59^ This fear further discourages Chinese individuals from openly discussing sexuality-related topics. Further research is required to specially explore the sexual experiences of Chinese HSCT survivors specifically. Addressing this issue is essential for providing holistic, person-centered care and ensuring that all aspects of survivors’ health, including sexual well-being, are adequately supported.

## Conclusions

This qualitative study offers novel insights into the subjective experiences of body image changes among Chinese patients with hematological cancers following HSCT within Chinese cultural context. The multifaced construct of body image was evident in HSCT survivors, with changes experienced as a dynamic process of body acceptance. Negative body image and struggles arising from identity negotiations warrant greater attention and timely intervention. The underlying mechanisms linking body image changes to shifts in self-identity, and the process of body image acceptance deserves further exploration. Additional studies are needed to investigate the prevalence of body image distress, its influential factors, the development of lifestyle guidelines, and the construction of a holistic positive body image model.

## CRediT authorship contribution statement

**Jia Yin Ruan**: Conceptualization, Methodology, Data collection, Data analysis, Writing – Original Draft, Writing – review and editing. **Lingxiang Lu**: Data collection, Writing – Review & Editing. **Wing Fai Yeung**: Data analysis, Writing – Review & Editing. **Ying Qian**: Writing – Review & Editing. **Mary-Ellen Brierley**: Writing – Review & Editing. **Yim Wah MaK**: Writing – Review & Editing. **Yiyu Zhuang**: Supervision, Methodology, Resources, Writing – Review & Editing. All authors approved the final version for submission.

## Ethics statement

This study was approved by the Medical Ethical Committee of Sir Run Run Shaw Hospital, Zhejiang University School of Medicine (Approval No. 20210104-30) and was performed according to the 1964 Helsinki Declaration and its later amendments or comparable ethical standards. All participants provided written consent.

## Data availability statement

The data that supports the findings of this study are available from the corresponding author, YZ, upon reasonable request.

## Declaration of generative AI and AI-assisted technologies in the writing process

No AI tools/services were used during the preparation of this work.

## Funding

This study received no external funding.

## Declaration of competing interest

The authors declare no conflict of interest.
